# Making Data Sharing Count: A Publication-Based Solution

**DOI:** 10.3389/fnins.2013.00009

**Published:** 2013-02-06

**Authors:** Krzysztof J. Gorgolewski, Daniel S. Margulies, Michael P. Milham

**Affiliations:** ^1^Max Planck Institute for Cognitive and Brain SciencesLeipzig, Germany; ^2^Center for the Developing Brain, Child Mind InstituteNew York, NY, USA; ^3^Nathan S. Kline Institute for Psychiatric ResearchOrangeburg, NY, USA

**Keywords:** data paper, data sharing, credit assignment, data quality, peer review

## Abstract

The neuroimaging community has been increasingly called up to openly share data. Although data sharing has been a cornerstone of large-scale data consortia, the incentive for the individual researcher remains unclear. Other fields have benefited from embracing a data publication form – the data paper – that allows researchers to publish their datasets as a citable scientific publication. Such publishing mechanisms both give credit that is recognizable within the scientific ecosystem, and also ensure the quality of the published data and metadata through the peer review process. We discuss the specific challenges of adapting data papers to the needs of the neuroimaging community, and we propose guidelines for the structure as well as review process.

## Introduction

Recent years have witnessed renewed calls for data sharing^1^ in neuroimaging (Chicurel, 2000; Van Horn et al., 2001; Van Horn and Toga, 2009; Visscher and Weissman, 2011; Milham, 2012; Poline et al., 2012), with inspiring demonstrations of the benefits of community-level sharing (Marcus et al., 2007b; Jack et al., 2008; Biswal et al., 2010; ADHD-Consortium, 2012; Mennes et al., 2012; Nooner et al., 2012). Concomitant advances in the necessary software infrastructures have increased the sense of readiness for the next era of community-wide sharing. In order to maximize the potential of these advances, we need to consider how the flagship of scientific culture – peer-reviewed articles – can actively participate in standardizing, disseminating, and ultimately crediting individual researchers for their sharing of data.

Given the continued challenges of “publish or perish,” tenure, and funding in a worsening economic and funding environment, the cost-benefit ratio has become central to any discussion of sharing (Fienberg et al., 1985; Poline et al., 2012). At the moment, it may be unclear to individual researchers if the community-wide benefits outweigh the personal cost of preparing and curating data for public distribution. Even preparation of fully analyzed and published data sets can take many hours of refinement (e.g., careful description of the data) and review prior to distribution – a cost that is often discussed in terms of lost scientific productivity for the individual (Tenopir et al., 2011). Consciously or unconsciously, such logistical challenges are often compounded by fears of embarrassment related to exposition of data errors or poor handling practices. Potentially most worrisome to junior and pre-tenure investigators are the fears of losing the competitive advantage associated with the sharing of their data – and all this without offering a clear benefit.

Numerous models have attempted to incentivize participation in data sharing, with varying success (NIH, 2003; Wellcome Trust, 2003; Organization for Economic Co-operation and Development, 2007; Birney et al., 2009; Contreras, 2011). Arguably most prominent among them is the creation of data sharing consortia – groups of researchers sharing similar interests that agree to combine their efforts. Consortiums can arise either pre- or post-data acquisition. Successful in fostering the aggregation of large-scale datasets and collaboration among investigators, consortia are not without their challenges (Singh and Daar, 2009). In particular, individual investigators may still struggle to gain recognition relative to the larger consortium – a reality that can be particularly problematic when contributions among members are not equal. Moreover, while examples of consortiums that openly share their data have emerged, this is not the norm, limiting the impact of current consortium models. Funding institutions have attempted to build on the model by forming well coordinated, data generating consortia explicitly for the purpose of generating and openly sharing data. The Human Connectome Project (HCP), Biomedical Informatics Research Network (BIRN), and Alzheimer’s Disease Neuroimaging Initiative (ADNI) are examples of funded, coordinated consortia. Unquestionably successful in the generation the highest quality data and innovative technologies, the exorbitant costs of these efforts (e.g., ADNI: $69 million, HCP: $40 million) limit both their scope and ability to be scaled to the larger community’s needs.

Central to this discussion is whether the data generator receives credit through authorship in publications by data users. In this regard, the field has noticed a clear divide, with consortia such as ADNI requiring explicit co-authorship on any manuscript generated with their data (ADNI, 2012), while others such as the 1000 Functional Connectome Project (FCP) and the International Neuroimaging Data Sharing Initiative (INDI) requiring only acknowledgment (Mennes et al., 2012). On an individual level, researchers will often require co-authorship to share their data with others. Although justifiable, a number of complexities arise when explicit requests for co-authorship are made. For example – how many co-authors are justifiable? On how many publications should the data generating co-authors be included? Is it appropriate for an individual to receive credit for neuroscientific or methodological innovations when they did not contribute to beyond data generation? Furthermore, there is no precedent for how to handle a situation in which the data user and data generator disagree on methodologies or data interpretation.

**Figure 1:**
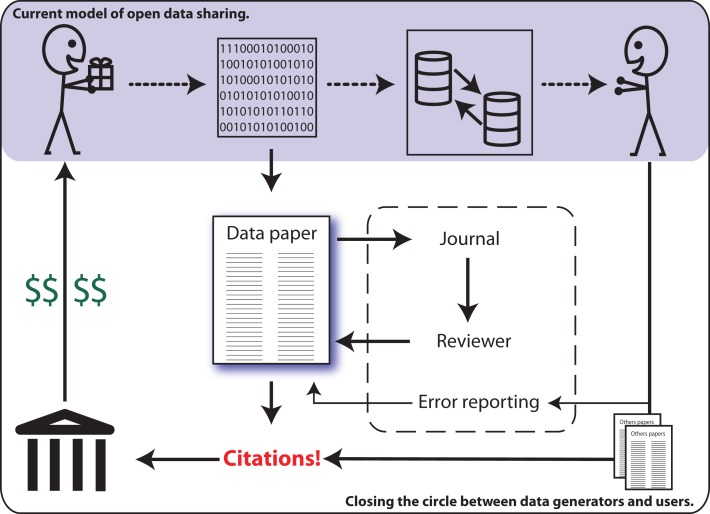
**The current model of data sharing (*top*) offers limited practical return for the data generator (*top left*)**. Data papers (*center*) enable researchers to publish and be cited for their efforts, thereby providing a clear mechanism for professional recognition (*left*).

In the search for solutions, some imaging researchers are calling for consideration of “data papers” as a means for increasing the potential benefits of data sharing for the individual investigator (Breeze et al., 2012). This builds upon the model employed by other scientific disciplines, such as genetics, where researchers are able to publish original articles in peer-reviewed journals that describe and characterize publicly shared data sets (Newman and Corke, 2009; Chavan and Penev, 2011). Similar to the Section “Methods” of a traditional research article, a data paper describes the data acquisition process, though with greater detail, and inclusion of a discussion of the rationale, motivation, and considerations regarding experimental design. Data papers do not provide any analysis nor results. The review process ensures the appropriateness and quality of methods employed for data acquisition, as well the completeness of descriptions provided. Data papers allow the data generating researchers to obtain publication credit for their work (Chavan and Penev, 2011), which is both citable by other investigators and appropriate for consideration in determinations of metrics of success (e.g., H-Index) – see Figure 1.

Despite the fact that this form of publishing data has been common in other fields (Ecological Archives: Data Papers, Supplements, and Digital Appendices for ESA Journals^2^, Earth System Science Data^3^, CMB data papers^4^, BMC Research Notes: Data Notes^5^, International Journal of Robotics Research)^6^, to date, only one neuroscience-dedicated publication venue has an article format dedicated to data papers: the journal *Neuroinformatics* (De Schutter, 2010; Kennedy et al., 2011). At present, there is no uniform format or hosting mechanism for published data. Non-neuroimaging journals can also publish data papers, with the ability to host data (i.e., *GigaScience*), but there is no uniform standard, nor minimal requirements, for data specification or format.

In sum, while the concept of data papers is of clear intrigue to the imaging community, the infrastructure for supporting their publication and dissemination is lacking. We feel this need from the experiential vantage of both data generators and users, and from having played key roles in the FCP/INDI consortia. In bringing these perspectives to bear on the proposed aims of data papers, the goals of the present work are twofold: First, we explore the mechanics of data papers and provide minimum standards with the hope of making the data paper concept more concrete and tangible for the community. Second, we review open questions and potential pitfalls for data papers, and offer solutions toward making data sharing appropriately rewarding at all levels of participation.

## Data Paper Mechanics

### Minimum requirements for data specification

Comprehensive and detailed specification of data samples is a prerequisite for the utility of data papers to be realized. Data papers should provide the data user with the information required to understand the data set at a level of detail known to the data generators. Without such requirements for thoroughness in description of the data generation process, we run the risk of misunderstood, and thus misused data sets (Gardner et al., 2003). Issues of data transparency could be resolved by specifying the minimum requirements for data sample description, which could then serve as a guide for the review process. Here we provide a brief overview of the minimum requirements to be addressed:

- Study Overview
  ○ Explicit goals for creation of data sample
  ○ Guiding principles in study design
- Participants
  ○ Sample size
  ○ Recruitment strategy
  ○ Inclusion and exclusion criteria
  ○ Sample demographics and characteristics
  ○ Informed consent methodology
- Experimental Design
  ○ Study type (e.g., cross-sectional vs. longitudinal)
  ○ Study timeline
  ○ Study workflow
  ○ Outline of scan session(s)
  ○ Task and stimulus descriptions, code used for presentation (when applicable)
  ○ Instructions given to the subjects
  ○ Description of data not included in shared data sample
- Phenotypic Assessment Protocol
  ○ Demographics
  ○ Phenotypic assessment protocol (when applicable)
  ○ Diagnostic assessments protocols (when applicable)
  ○ Qualifications of research staff collecting data (including measures of rater reliability when appropriate)
- Scan Session Details
  ○ MR protocol specification describing the order, type, purpose, and acquisition parameters for each scan
  ○ Conditions for each scan [e.g., eyes open/closed for resting state fMRI (R-fMRI), watching movie, or listening to music for structural, stimuli for task fMRI – see experimental design]
- Data Distribution
  ○ Distribution site
  ○ Distribution type (database, repository, local ftp)
  ○ Imaging data formats (e.g., NIFTI, DICOM, MINC)
  ○ Imaging data conventions (e.g., neurological vs. radiological)
  ○ Phenotypic data key
  ○ Missing data
  ○ License

### Specification of design motivation and rationale

Successful use of shared data requires a clear and comprehensive description of the study’s motivation and design. Similarly, it is important to be aware of alternative designs that were considered, and the rationale for their rejection. Without such knowledge, user analyses and interpretations may be susceptible to biases (e.g., recruitment and sampling) that limit the validity or generalizability of findings. For example, in studies of psychiatric populations characterized by heterogeneity (e.g., Attention Deficit Hyperactivity Disorder, schizophrenia, autism), some investigators may bias their samples in favor of one behavioral profile or clinically defined subtype over another due to the specific hypotheses to be tested, or relative ease of recruitment (e.g., when one subtype has a higher prevalence than another). Others may take all volunteers (i.e., opportunistic sampling) or use an equal representation of subtypes, either treating the population as unitary or analytically accounting for the heterogeneity.

Increasingly, researchers are sharing data that was obtained as part of a larger study. Although well-justified and encouraged, biases can be introduced by the other data obtained as part of the effort, and need to be taken into account. An obvious example comes from R-fMRI studies, where scans are often “tacked onto” the end of ongoing task-based activation studies. Failure to provide users with information about task-activation scans included prior to the occurrence of a resting scan in a given study can result in systematic biases of R-fMRI metrics related to the nature of the task performed (McIntosh et al., 2003; Hampson et al., 2004; Rissman et al., 2004). Similarly, if a scan is obtained at the beginning vs. the end of an imaging session, notable variability can be introduced; users need to understand the decisions made in the experimental design process to fully appreciate their impact. Each data paper should specify the motivation that has driven the researchers to collect the data. Additionally, even if only a subset of the data would be released (for example just the R-fMRI data out of a sequence including DTI and a few task scans) information about the non-disclosed data (without providing the data itself) should be included. Reviewers should be advised to double check suspiciously short sequences in context of the study motivation and make sure that the authors are providing all the contextual information. Additionally all papers published up to date that were using the presented data should be referenced. This will allow future users of the data to know what results should they expect in case of a replication.

### Data-release type

Data sharing and open science are two related but distinct phenomena. Individuals can choose to share their data with a limited set of collaborators or the broader community. Even for datasets intended for open access, it is possible that data usage agreements must first be used to protect participant privacy. Importantly, publication of a data paper regarding a restricted dataset could share useful insights into experimental design and/or potentially motivate members of the scientific community to approach the generating authors and seek collaboration (Gardner et al., 2003). From a pragmatic point of view, authors who choose to publish data papers and share with no one will have a low likelihood of citation, unless they share potentially valuable design decisions; in contrast, open access datasets stand a greater likelihood of frequent citation. Given these considerations, it is our proposition that data publications should not be limited to open access datasets. However, it is of utmost importance that authors explicitly state their data sharing policy and abide by it.

### Data-release timetables

In the past, authors in journals such as “Science”^7^ and “Proceedings of the National Academy of Sciences” have had statements in publications stating the data included in the work would be shared, though without clear mechanisms or timetables for sharing in place (Cozzarelli, 2004). When promised, failure to share data in a timely manner can undermine the integrity of the process and trust of the user base. Data papers should be explicit in the description of when and how their data will be shared (e.g., data to be shared at time of data paper publication, open access after a specified embargo date, restricted access). Regardless of the sharing policy, reviewers should have access to the data to verify its congruence with the report provided in the submitted manuscript, as well as readiness for data sharing (when applicable). Inspection of data intended for sharing is especially important when data are being hosted by venues other than the journal or well-established sharing mechanisms.

### Ethics board requirements

Despite calls for consensus among ethics board regarding standards for sharing, marked variation remains. The FCP/INDI efforts provide a clear example of the variability among ethics boards in their decision-making. Built upon the premise of full anonymization of data in compliance with the 18 protected health identifiers specified by the Health Insurance Portability and Accountability Act (HIPAA) privacy rules, the FCP/INDI efforts do not require prior consent by participants for sharing. While the vast majority of ethics boards supported this decision, either deeming the de-identified data no longer be human research data, or arguing the benefits outweigh the minimal risks of sharing, some did require re-consenting. Importantly, not all data can meet the standards of the HIPAA privacy rules (e.g., community-ascertained samples such as the NKI-Rockland Sample, where the county of residence is known for all participants). Even if fully de-identified, concerns can exist for the sharing of data sets with comprehensive medical information or potentially incriminating information (e.g., substance use), increasing the risks associated with data sharing (Ohm, 2010). Additionally one has to consider differences in privacy policies between countries. Data papers should explicitly state the nature of the consenting process for sharing (in the context of legislations of the country it was acquired in), verify the appropriateness for sharing and compliance with local ethics board requirements, and include a brief assessment of risk in the case of privacy breach.

### Authorship/crediting

A major motivating force for data papers is to ensure that all parties involved in a research endeavor receive proper credit. Unfortunately, a common tension in the preparation of any research report is the determination of individuals meriting authorship (Pearson, 2006). Given the increased acquisition and analytic demands of imaging studies, author lists have rapidly increased in size. Although justifiable, as proper crediting of contributions is an essential part of the scientific process, such increases can raise concerns from individuals about “getting lost in the list” (Wyatt, 2012). Such concerns can pressure investigators to “demote” authors from the list. Data papers offer those most directly involved in the design and generation of data the opportunity to receive proper recognition, separate from data users. Simultaneously, they can serve to reduce the burden of recognition in more analytically oriented papers. Additionally, space in the Section “Methods” of a research article (often relegated to supplementary materials) can be saved by referencing an appropriate data paper instead of describing the acquisition process in full.

### Review guidelines for data papers

Central to the success of data papers, is to conduct high quality peer review that is tantamount to more traditional research report formats. Failure to ensure that reporting of the goals, design, and description for the sample being reported on, or the quality control and fidelity checks employed during acquisition will rapidly undermine the data sharing process and the credibility of data papers. Similarly, if a reviewer is not able to directly review the data distribution prepared by the author and verify the readiness of the shared sample for distribution, the process will be compromised. It must be noted that it should not be expected of the reviewer to manually check each data item to be shared – such an expectation would likely discourage reviewers from participating in the data paper process. Reviewers can be expected to assess the appropriateness of described data preparation and distribution mechanisms for sharing, as well as the venue(s) selected for sharing and the readiness of the reported data for sharing upon acceptance of the data paper. Individuals selected to review a data paper should be capable of evaluating study design and collection for a procedures for a given sample, as well as a person versed in sharing procedures that can evaluate venue. Explicit data paper standards and checklists should be provided to reviewers by journals to ensure a minimum standard for data papers.

### Data error correction reporting

As demonstrated by the various FCP and INDI releases, the need for data updates and corrections post-release is a reality^8^. Data sharing allows outside investigators to audit the data in ways that the original data owners may not have ever considered, at times revealing unseen errors in the data (e.g., occasional left-right flips in the initial FCP release). In this regard, data papers cannot be considered definitive at time of publication, as errors undoubtedly will be uncovered over the course of time. Failure to update the data user population can lead the propagation of erroneous findings. A fast and easy way to submit issues and corrections should be put in place. Small corrections could be achieved by a system of comments (see Frontiers), but more substantial revisions would require editorial work. Importantly, the reporting of data error corrections should be strongly encouraged, and facilitated by the journal. Usage of corrigendum (preferred) or addendum mechanisms for error correction updates to data papers (rather than erratum) can avoid unintended discouragement of authors in their reporting of errors. Importantly, paper updates do not replace the need for data to be distributed with an up to date log that describes all changes and corrections made since the initial publication.

## Open Questions and Pitfalls

### Open access vs. restricted for data papers?

A relevant question is whether publication in a subscription journal works against the collaboration-promoting goals of data papers. Although a valid point, such an assertion likely minimizes the complexities of publication, including cost-sharing. For example, while open access journals are increasing touted for the removal of barriers to readers, the cost is shifted to authors, which can be prohibitive for some. Also, many subscription journals have open access options, equating them to open access journals if authors are willing to pay the fee. Additionally, as already noted, data papers should not necessarily be limited to open access datasets. In the end, it would appear that open access publications would gain maximal exposure and citations (Eysenbach, 2006), motivating authors to pursue this venue; though this remains an empirical question. No matter what type of access authors decide to use for their data they should be explicit about it in the data paper. An intriguing option is to have journals, funding agencies, or institutions incentivize open access data papers through the waiving of open access publication fees or provision of a stipend to cover open access costs in the case of open data sharing; although attractive, the feasibility is unclear.

### Centralized, federated, or independent data hosting platforms?

Currently there is no centralized mechanism for hosting data, and it is unclear whether or not the field would be willing to accept such an entity. Efforts are currently underway to create the possibility of a federation among data sharing resources (e.g., INCF dataspace)^9^, though at present, these are in the development stage. Currently, an individual researcher willing to share his data can put them into one of the existing data repositories or databases^10^^,^^11^^,^^12^, or host the data his/herself. With respect to self-hosting, maintaining access to data sets is neither cheap nor easy; additionally, continuity of access to data can be tied to the employment status of the author. On the other hand, external databases and repositories are reliant on the procurement of funding from grant agencies and/or foundations to maintain continuity of service; as such, they can not maintain guarantees of indefinite data hosting. Of note, journals such as GigaScience have presented an alternative model, in which the journal itself hosts data, paid for by submission fees. Regardless of the hosting entity, one consideration is the inclusion of Digital Object Identifiers (DOIs) that exist independently of the internal journal and can be updated if and when the location of hosted data changes (Paskin, 2005; Brase et al., 2009), thereby facilitating continuity. It is important to note that when two or more venues host the same dataset (Mennes et al., 2012), or a dataset is rebroadcast, synchronization of version/change-logs across databases is desirable, but can become complicated and disparities may arise. In this regard, we once again highlight the need to include revision logs with all datasets as a potential solution.

### Can neuroinformatics tools facilitate the generation and review of data papers?

Although not a necessity to initiate the process of data papers, over time, neuroinformatics tools hold great potential in facilitating rapid generation and review of data papers, as well as the preparation of datasets for sharing. With respect to the generation of data papers, sophisticated data basing systems such as COINS (Scott et al., 2011), LONI Image Data Archive, LORIS (Das et al., 2011), XNAT (Marcus et al., 2007a), and HID (Ozyurt et al., 2010) hold the potential to facilitate authors in the automated generation of summary statistics, characteristics, and quality control for imaging data (e.g., motion-metrics, image quality, signal dropout) and phenotypic data (e.g., range checking) in a given sample, as well as minimize the potential for errors. Semi-automated solutions can be developed to facilitate rapid review of data distribution readiness by reviewers (Stöcker et al., 2005). It is not our belief that the generation of data papers should wait for neuroinformatics to “catch up”; rather, it is our hope that the process will further motivate the allocation of resources to the generation of tools capable of increasing the accuracy and efficiency of the data paper process.

### One data paper or more?

While we have aimed to describe how data papers could benefit the neuroimaging research community, they are not immune to more general problems that are present across the current publishing landscape. For instance, a common challenge for any large-scale effort is determining whether to report the work as one single project, or to break it into two or more smaller studies for the purpose of increasing the speed or number of publications. Similar concerns may arise in data papers, where researchers may be tempted to divide a design into pieces, and to report on sub-cohorts separately. Such practices should be discouraged at all levels of the publication process, as only complete data publication provides a clear understanding of the intended experimental design and decisions made.

## Making Data Papers Count: Funding and Promotion

A key concern is that data papers may fall outside what is typically valued by the academic community as “scholarly” contributions. Appropriate citation of data papers can make it possible for researchers to receive credit for data sharing via publication-based metrics of impact. However, creation of alternative metrics that directly assess data generation productivity via data paper publication and citation will likely prove to be more effective in drawing attention to the value of data sharing on biosketches and curriculum vitae, which are currently deficient in this regard. In the bigger picture, funding, and academic institutions will have to work on the development of formal policies for promoting and rewarding investigators generating data of use to the scientific community. Some mechanisms do currently exist. For example, the “broader impact” portion of National Science Foundation (NSF) applications allows reviewers to reward those proposing designs of value to the larger community, beyond their own goals. Data papers can help provide a track record to support the promises of investigators in sharing the data generated. Additionally, inclusion of funding acknowledgments in data papers provides researchers an opportunity to gain proper recognition for their efforts, and will help researchers document compliance with data sharing mandates. Although helpful, these mechanisms are not sufficient to fully reward researchers for their efforts in data sharing in funding decisions and do little to assist with major areas of concern, such as tenure decisions and fulfillment of departmental obligations.

## Summary

Data papers hold the potential to provide data generating researchers the much deserved recognition for their efforts in study design, execution, and maintenance, while at the same increasing their motivation to share and collaborate with others through the publication process. The present work detailed minimum requirements to ensure the completeness and utility of data papers, and provided initial insights into controversies or questions that may arise for authors, reviewers, publishers, and data users in the process. A key remaining challenge is the reform of academic and funding institution practices to increase recognition of the need for, and scientific merits of generation of high quality data for the field by those best suited for the task. We hope that the development, development, and refinement of neuroinformatics tools will facilitate and further motivate the data paper process in the future.

## Conflict of Interest Statement

The authors declare that the research was conducted in the absence of any commercial or financial relationships that could be construed as a potential conflict of interest.

## Footnotes

^1^All data sharing efforts discussed in this article concern raw data; derived data poses unique issues beyond the scope of the current proposal.

^2^http://www.esapubs.org/archive/default.htm

^3^http://www.earth-syst-sci-data.net/

^4^http://lambda.gsfc.nasa.gov/outreach/recent_papers.cfm

^5^http://www.biomedcentral.com/bmcresnotes/ifora/?txt_jou_id=4005&txt_mst_id=104807

^6^http://ijr.sagepub.com

^7^http://www.sciencemag.org/site/feature/contribinfo/prep/gen_info.xhtml

^8^http://www.nitrc.org/forum/forum.php?thread_id=3169&forum_id=1735

^9^http://www.incf.org/resources/data-space

^10^http://central.xnat.org

^11^http://fcon_1000.projects.nitrc.org

^12^http://OpenfMRI.com
